# Supplementary material for: Modeling the Impact of Interventions on an Epidemic of Ebola in Sierra Leone and Liberia
Source: PLoS Curr. 2014 Oct 16;6:ecurrents.outbreaks.fd38dd85078565450b0be3fcd78f5ccf. [Version 1] doi: 10.1371/currents.outbreaks.fd38dd85078565450b0be3fcd78f5ccf (PMC4399521; doi:10.1371/currents.outbreaks.fd38dd85078565450b0be3fcd78f5ccf)
Supplement: Related texts

# Reply

**Published:** 2014-10-21

**Authors:** testing123321

Are the weights in Figure 1 incorrect? The authors weight the rates by probability, but they should be weighting the average time spent in each compartment (i.e. 1/rate) and then inverting this again. For a simple R example, let us take a compartment spawning two options, one with rate 1/4 and one with rate 1/1, each with 50% probability: 1/mean(c(rexp(1000000,rate=1/4),rexp(1000000,rate=1/1))) This gives a value of 0.4. Which is equal to 1/(4*0.5+1*0.5). This is NOT equal to 1/4*0.5+1/1*0.5=0.625
